# A Native-Like Corneal Construct Using Donor Corneal Stroma for Tissue Engineering

**DOI:** 10.1371/journal.pone.0049571

**Published:** 2012-11-15

**Authors:** Jing Lin, Kyung-Chul Yoon, Lili Zhang, Zhitao Su, Rong Lu, Ping Ma, Cintia S. De Paiva, Stephen C. Pflugfelder, De-Quan Li

**Affiliations:** 1 Ocular Surface Center, Cullen Eye Institute, Department of Ophthalmology, Baylor College of Medicine, Houston, Texas, United States of America; 2 Department of Ophthalmology, the Affiliated Hospital of Qingdao University Medical College, Qingdao, China; 3 Department of Ophthalmology, Chonnam National University Medical School and Hospital, Gwangju, South Korea; University of Reading, United Kingdom

## Abstract

Tissue engineering holds great promise for corneal transplantation to treat blinding diseases. This study was to explore the use of natural corneal stroma as an optimal substrate to construct a native like corneal equivalent. Human corneal epithelium was cultivated from donor limbal explants on corneal stromal discs prepared by FDA approved Horizon Epikeratome system. The morphology, phenotype, regenerative capacity and transplantation potential were evaluated by hematoxylin eosin and immunofluorescent staining, a wound healing model, and the xeno-transplantation of the corneal constructs to nude mice. An optically transparent and stratified epithelium was rapidly generated on donor corneal stromal substrate and displayed native-like morphology and structure. The cells were polygonal in the basal layer and became flattened in superficial layers. The epithelium displayed a phenotype similar to human corneal epithelium in vivo. The differentiation markers, keratin 3, involucrin and connexin 43, were expressed in full or superficial layers. Interestingly, certain basal cells were immunopositive to antibodies against limbal stem/progenitor cell markers ABCG2 and p63, which are usually negative in corneal epithelium in vivo. It suggests that this bioengineered corneal epithelium shared some characteristics of human limbal epithelium in vivo. This engineered epithelium was able to regenerate in 4 days following from a 4mm-diameter wound created by a filter paper soaked with 1 N NaOH. This corneal construct survived well after xeno-transplantation to the back of a nude mouse. The transplanted epithelium remained multilayer and became thicker with a phenotype similar to human corneal epithelium. Our findings demonstrate that natural corneal stroma is an optimal substrate for tissue bioengineering, and a native-like corneal construct has been created with epithelium containing limbal stem cells. This construct may have great potential for clinical use in corneal reconstruction.

## Introduction

Ocular surface diseases with corneal epithelial stem cell deficiency, such as Stevens-Johnson syndrome, chemical, thermal and radiation injuries, extensive microbial infection, and inherited disorders such as aniridia, are sight threatening and often cause blindness (see review [1]). Transplantation of a corneal limbal graft that contains corneal epithelial stem cells (also referred to as limbal stem cells) can help to restore vision. Although corneal transplantation has achieved clinical success, there is an increasing shortage of corneal donors worldwide. There are over 10 million global blinding patients caused by corneal disease due to the lack of cornea donors for corneal transplantation. Corneal tissue engineering is becoming an important discipline that holds great promise for corneal transplantation to treat the blinding corneal diseases [2]–[4].

The first clinical success of the cultivated corneal epithelial transplantation was noted in a report of two patients published by Pellegrini and colleagues in 1997 [5]. After this initial report, more investigators reported transplantation of in vitro cultivated corneal epithelium on the substrate carriers using amniotic membrane [6]–[8], fibrin [9], [10], and collagen hydrogel [11]. All these substrates have great impact for clinic corneal transplantation. However, in establishing corneal tissue equivalents for transplantation, there is still need to develop new optimal structures. Ideal substrates should be optically transparent, should support corneal epithelial progenitor cell survival, should promote cell differentiation to form a native-like stratified epithelium, and should serve as a carrier for easy transplantation to patients.

Based on the Eye Banking Statistical Report from the Eye Bank Association of America, in 2009, among a total of 107,289 donor globes and corneas, procured by 78 U.S. eye banks, only 55.72% of donor corneas were used for transplantation. The defective corneas, including those with poor endothelial health, were over 20,000 donor corneas per year and accounted for 38.6–46.1% of total donor corneas. We believe that these defective corneas can serve as an invaluable resource for obtaining corneal stroma as a native substrate for cornea tissue engineering. We hypothesize that the natural corneal stroma can be utilized as a new optical substrate to promote regeneration of a native like human cornea epithelium for tissue bioengineering and corneal reconstruction. The present study explored the utilization of natural donor corneal stroma in corneal tissue regeneration, and evaluated the phenotype, functional properties, as well as transplantation potential of the native-like corneal epithelium regenerated from limbal stem cells on the corneal stroma.

## Results

### Cultivation of Corneal Epithelium on Human Corneal Stroma from Limbal Epithelium Where Stem Cells Reside

**Figure 1:**
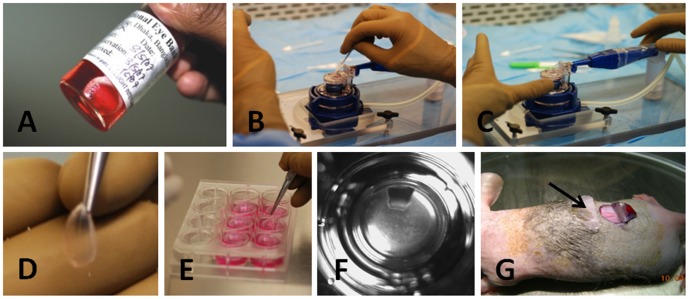
Regeneration of a native-like corneal construct using limbal explants and donor corneal stromal discs. **A.** Donor corneas stored in Optisol medium; **B.** Donor corneal epithelium was scraped; **C.** Horizen Microkeratome system used to fabricate corneal stromal lamella discs; **D.** A fabricated corneal stromal disc with 10–11 mm diameter and 200 µm thickness; **E.** The stroma disc was stored in culture medium; **F.** A fresh limbal explant cultured on the stromal disc that was placed in the culture insert; **G.** Xeno-transplantation of the corneal construct into the back skin of a nude mouse.

**Figure 2:**
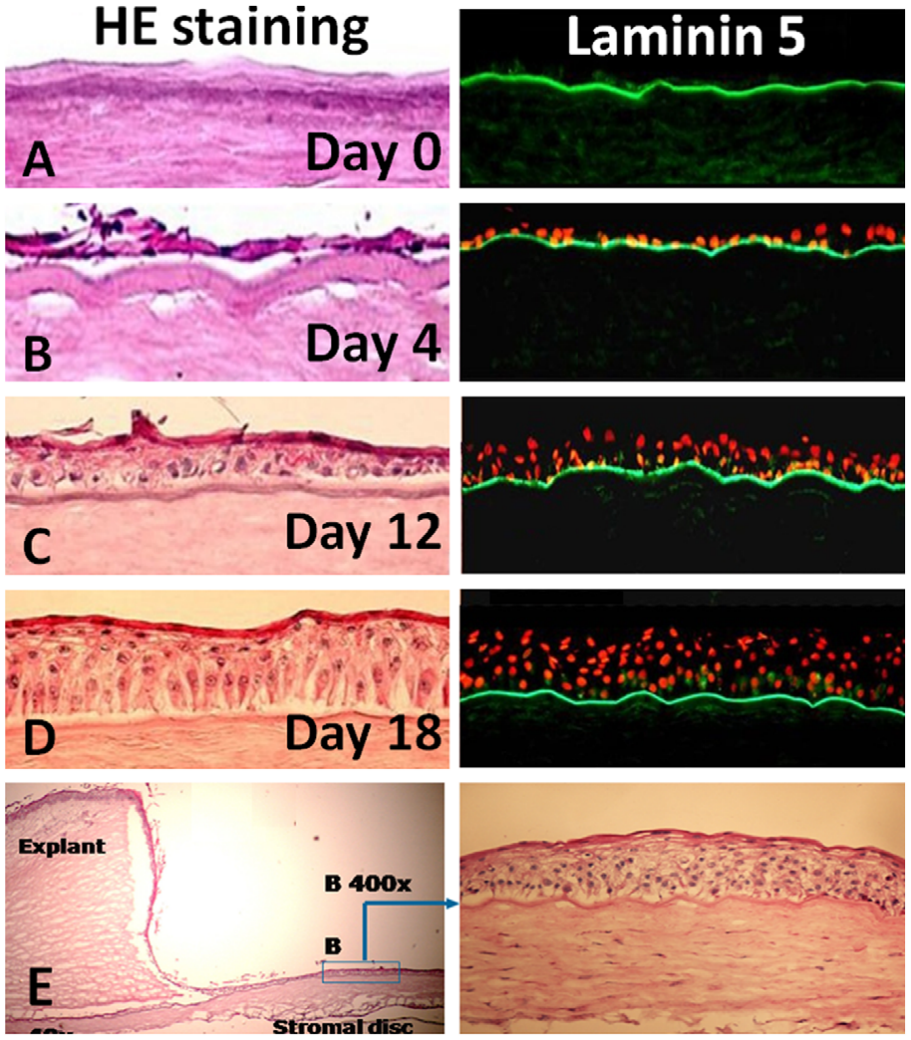
Growth of limbal epithelial cells on corneal stroma. **A.** The de-epithelial donor corneal stroma contained Bowman’s layer with entire basement membrane shown by HE and Laminin 5 staining; **B–E.** Limbal epithelial cells grew from one to multiple layers from a fresh limbal explant.

A total 62 of human corneal stroma discs were prepared by the Horizon Epikeratome system and used as substrate for limbal explants cultures (Fig 1 A–F). When stem cell-containing limbal explants cultured on corneal stromal discs, the human limbal epithelial cells (HLECs) grew earlier (initially at day 2–3), reached confluent more rapidly (in 10–12 days), and in all cases formed stratified multi-layer epithelia after air-lifting for 5–7 days, in comparison with the limbal explants grown on culture inserts alone as a control. An epithelium with 5–7 multi-layers was observed in all limbal explant cultures on the corneal stromal discs, which resembles the native-like human corneal epithelial morphology and structure in vivo, as examined by cross-sections of the cultures. As shown in Fig 2A by Hematoxylin and Eosin (HE) Staining and laminin 5 immunostaining, the prepared corneal stromal discs contain an intact basement membrane that overlies Bowman’s layer after the donors’ corneal epithelium has been completely removed. HLECs grew well on the stromal discs from one layer in early days (Fig 2B) to form multi-layer epithelium in 2–3 weeks (Fig 2 C–E). The cells in the basal layer were polygonal and well-organised, and cells became more flattened in the suprabasal and superficial layers.

### Morphological Comparison of the Corneal Epithelia Generated on Different Substrates

**Figure 3:**
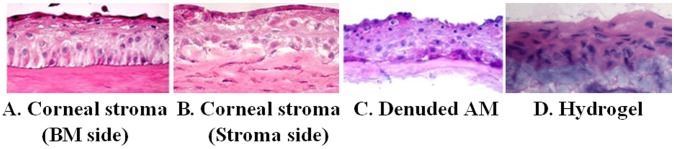
Morphology and structure of generated corneal epithelia on different substrates. **A.** on basement membrane (BM) side of cornel stroma; **B.** on stromal side of cornel stroma; **C.** on denuded amniotic membrane (AM); **D.** on a hydrogel.

We compared the corneal epithelia generated on the both sides, the basement membrane or stroma side of the donor corneal stroma, as well as on the amniotic membrane, and on the hydrogel. As shown in Fig 3, it appeared that the artificial corneal epithelium generated on the basement side of stromal disc showed the best morphology and structure similar to the native cornea structure in vivo. The cells at the basal layer were polygonal and well organized while the suprabasal cells were flattened like wing cells, and superficial layers were similar to apical cells in vivo. The results indicated that the intact basal membrane on the stroma Bowman’s layer is important to cultivate a native-like corneal epithelium, especially for the basal cell layer.

### Phenotype of the Epithelium Generated on Corneal Stromal Discs

**Figure 4:**
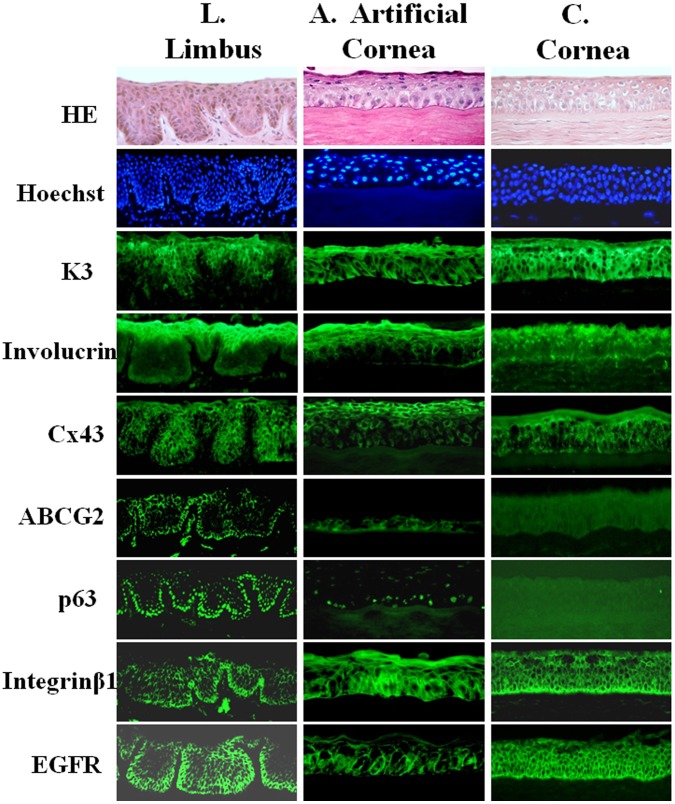
Phenotype of artificial corneal epithelium generated on donor stroma. Representative images of immunofluorescent staining for corneal epithelial markers (Cx43, K3, involucrin, ABCG2, p63, integrin β1 and EGFR) with Hematoxylin-eosin (HE) and Hoechst 33342 nuclear counterstaining on frozen sections of the artificial corneal construct (A), in comparison with those from donor tissues, limbus (L) and cornea (C).

In order to evaluate the phenotype of the epithelium cultivated from limbal explants on corneal stromal discs, we compared its expression pattern of corneal epithelial markers with that by the donor corneal and limbal epithelia. As shown in Fig 4 by immunofluorescent staining, this artificial epithelium expressed the corneal epithelial specific marker cytokeratin 3 (K3), the differentiation marker involucrin, and a gap junction protein connexin 43 (Cx43) at full or superficial layers (Fig 4A), the similar pattern to native corneal epithelium (Fig 4C). Interestingly, this artificial corneal epithelium shares some characteristics of human limbal epithelium in vivo. Their basal cells were partially positively stained by antibodies against limbal stem/progenitor cell markers ABCG2 and p63 (Fig 4A), which are usually expressed by limbal basal cells (Fig 4L) but negative in corneal epithelium in donor tissues (Fig 4C). The cells in the basal layer of artificial corneal epithelium were also more strongly stained by antibodies against stem/progenitor cell markers integrin β1 and EGF receptor (EGFR) than those in superficial layer.

### Regenerative Capacity of the Artificial Epithelium in an in vitro Wound Healing Model

**Figure 5:**
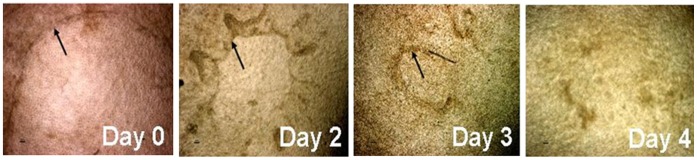
Phase images showed the healing capacity of wounded epithelium in the artificial corneal construct after a 4 mm diameter alkaline burn. Arrow points the edge of wounded area.

In order to evaluate the regenerative capacity of this artificial corneal construct, we created a wound healing model in vitro culture condition. A alkaline burn was made on these multi-layer epithelia generated on the 10 mm diameter stroma discs by a touch (5 seconds) with 4 mm diameter filter papers soaked with 1 N NaOH. The wounded areas began to heal on day 2 and completely healed in 4 days (Fig 5). This wound healing capacity was maintained after several repeated alkaline burns (data not shown).

### Xeno-transplantation of the Human Corneal Construct in Mice

**Figure 6:**
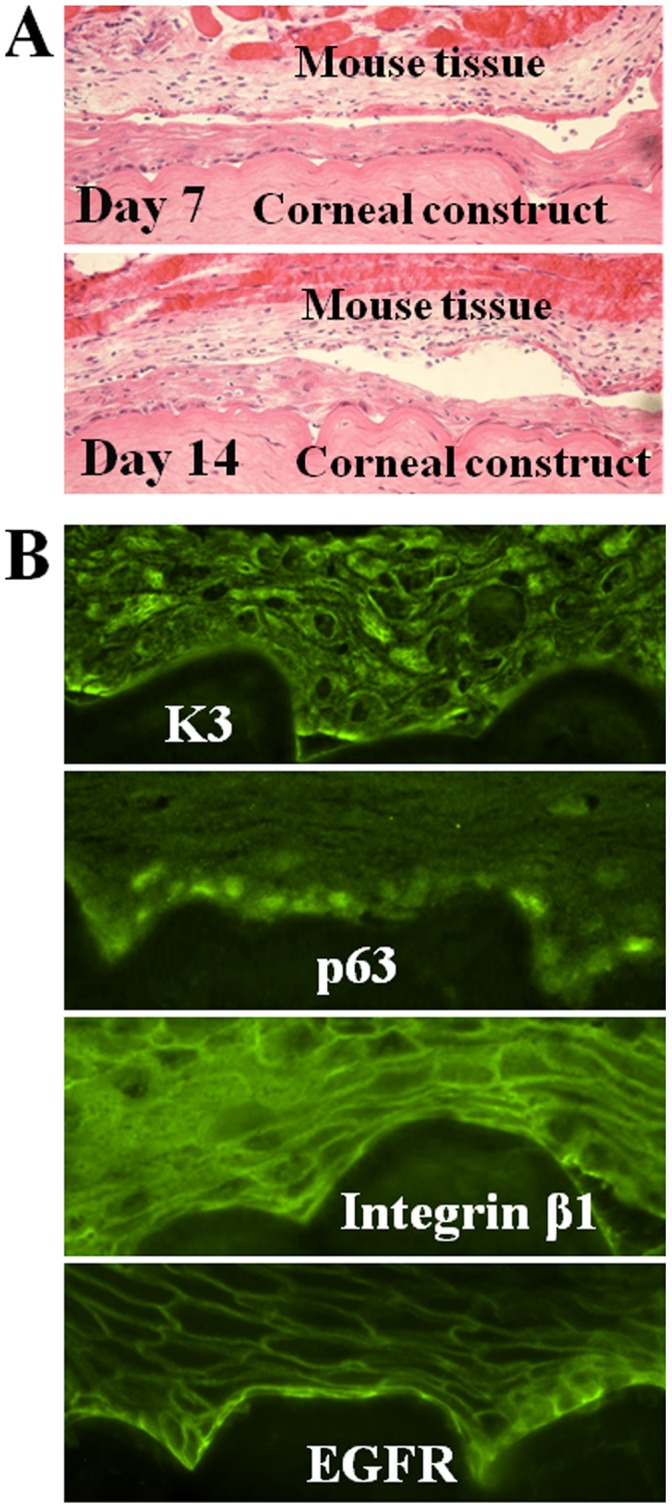
Xeno-transplantation of the artificial corneal construct in nude mouse back skin. A, Hematoxylin-eosin (HE) staining images (100×) showed that the corneal constructs survived and grew well in 14 days on nude mouse back. **B.** Immunofluorescent staining images (400×) showed that the K3, p63, integrin β1 and EGFR were expressed by the artificial corneal constructs transplanted in 14 days.

In order to evaluate the potential utilization of transplantation, the corneal constructs were xeno-transplanted into the back skin of NIH bg-nudxidBR nude mice. We observed that the epithelium was well survived after xeno-transplantion. The transplanted epithelium remained intact and multilayers after 7 and 14 days as evaluated by HE staining (Fig 6A). Immunofluorescent staining showed that corneal epithelial specific markers K3 was expressed by full layers of corneal epithelial cells while epithelial sterm/progenitor cell markers p63, intergrinβ1 and EGFR were strongly immunelocalized at the basal layer cells (Fig 6B). This phenotypic pattern was similar to pre-transplantation condition, suggesting that the transplanted corneal epithelium still shared some features of human limbal epithelium in expression of some progenitor markers. These results suggested that the epithelial cells of corneal construct survived well and still contained progenitor cells after transplantation.

## Discussion

Limbal stem cells and optical substrates are known to be the most important factors for corneal tissue bioengineering in regenerative medicine [3]. In the present study, human corneal stroma was used as a native optical substrate to reconstruct human corneas with stratified multilayer epithelium. The natural corneal stroma is a good substrate with two advantages: 1) providing appropriate scaffolds where the stem cells can proliferate and recapitulate native corneal structure and functions [12]; and 2) containing highly organized collagen lamellae that provides mechanical support and light diffraction properties appropriate for transparency [12], [13]. Our study suggests that the donor corneas that do not meet the criteria for direct clinical transplantation are a potential treasure for corneal tissue regeneration by using their natural stroma as a high quality optical substrate.

### Donor Corneal Stroma Served as a Native Substrate Supporting Corneal Epithelial Regeneration

In this study, we evaluated the growth of limbal tissue explants that containing limbal epithelial stem cells on 62 of donor corneal stromal discs that had been preserved in Optisol medium for 13–90 days. Recent studies have shown that collagen substrate stiffness influences relative levels of epithelial cell differentiation; and that the stiffness of corneal stroma decreases with depth [14], [15]. For the consistency of stromal quality, we only use the anterior corneal stroma with 200 µm thickness, and make only one stromal disc from each donor cornea. The morphology and structure of the epithelium cultivated on the basement membrane side of corneal stromal discs are well organized when compared with epithelium grown on the stroma side of corneal stroma, amniotic membrane or hydrogel (Fig 3). The intact basal membrane is an important feature of the donor corneal stroma for supporting native-like corneal epithelial regeneration. Laminin 5, a ligand of integrins α3β1 and α6β4, is essential constituent components of the lamina lucida of the corneal basement membrane. The positive staining of Laminin 5 in Fig 2 showed the integrated basement membrane of our native-like corneal equivalent. Like other basement membranes in the body, the corneal basement membrane anchors epithelial cells and provides positional information for healing, repair and tissue regeneration [16], [17]. The well stratified and organized corneal epithelium is importance in vision and in protecting the eye against external injuries. We found that this well organized native-like corneal epithelial structure is not often seen in corneal epithelia cultivated on other substrates such as temperature-responsive culture surface [4], human amniotic membrane [7], [18] and hydrogel. Our results showed that corneal stroma discs with integrated basement membrane promote growth and stratification of limbal epithelial cells.

### Corneal Epithelium Generated from Limbal Tissue on Corneal Stroma Contains Limbal Stem/Progenitor Cells

The qualified corneal construct should express keratins that ensure its stability and integrity, which allow the anchorage between the epithelium and the basement membrane, as well as between epithelial cells themselves. K3, as a specific marker for corneal epithelium, is mainly located in the all layers of corneal epithelium and superficial and suprabasal layers of limbal epithelium, but is not expressed by undifferentiated limbal stem cells at the basal layer of limbal epithelium [19]. Involucrin, a precursor of the cornified envelop [20], and Cx43, a gap junction protein [19], [21], are considered as differentiation markers, which are not expressed by the basal cells of limbal epithelium. Consistently, our result showed that the immunoreactivity of K3, Cx43 and involucrin was found in most layers of our corneal construct cultivated on donor corneal stroma (Fig 4). These findings suggest that this regenerated corneal epithelium possesses a similar phenotype to native corneal tissue.

Although the corneal epithelial stem cells have been identified in the limbus for more than two decades and many stem cell markers have been proposed, there is no single specific marker or definitive method that can identify the limbal stem cells to date [22]–[25]. Thus, we have characterized a unique phenotype of limbal epithelial basal cells that contain putative stem cells. By extensive evaluation of the proposed markers in our previous publications [19], [21], [26]–[32], we have characterized that the limbal stem/progenitor cells are small primitive cells expressing three patterns of molecular markers: (1) exclusively positive for p63, ABCG2, NGF, GDNF and TCF4 by a subset of basal cells; (2) relatively higher expression of integrin β1, EGFR, K19 and α-enolase by most basal cells; and (3) lack of expression of differentiation markers, E-cadherin, connexin 43, involucrin, K3 and K12. Based on this unique phenotype, we have further partially enriched the putative limbal stem/progenitor cells from donor limbal tissues and their cultures [21], [27]–[29].

Interestingly, we observed a unique phenomenon that our artificial corneal epithelium not only showed a phenotype similar to the typical human corneal epithelium but also shared some characteristics of human limbal epithelium in vivo when compared with phenotypes of donor corneal and limbal epithelial tissues (Fig 4). Limbal stem/progenitor cell markers ABCG2 and p63 are known to be exclusively expressed by basal cells of limbal epithelium, but not expressed by corneal epithelium [19]. However, we observed an expression of ABCG2 and p63 in certain basal cells of our artificial corneal epithelium. The stem/progenitor cell markers integrinβ1 and EGFR were also more strongly immunolocated in the basal layer than in suprabasal layer of the artificial corneal epithelium. The high expression of integrin β1 indicates the strong adhesion of the cultured basal cells to specific extracellular matrix ligands [19]. The presence of high levels of EGFR might allow these cultured cells to be rapidly stimulated by growth factors to undergo cell division during development and following wounding. These findings demonstrate that the regenerated corneal epithelium shares some phenotype of limbal stem cells, suggesting it contains more stem/progenitor cells, an important feature for maintaining proliferation capacity and self-renewal.

To confirm the proliferation capacity and regenerative function of our corneal constructs, we performed an alkaline burn experiment as a wound healing model. As shown in Fig 5, the 4-mm diameter burn area of artificial epithelium on the basement side of stroma was completely healed in 4 days. This wound healing capacity was maintained after several repeated alkaline burns. The findings indicate the proliferative and regenerative capacity of the epithelium was maintained in this corneal construct.

### Transplantation Potential of the Corneal Constructs Fabricated with Stromal Discs

A final consideration of this study was to explore the clinical application of this native-like corneal construct. In order to evaluate the potential utilization for transplantation, the corneal constructs fabricated with stroma discs were transplanted into the back skin of NIH bg-nudxidBR nude mice. We observed that the epithelium well survived after xeno-transplantion at least for 14 days. The transplanted epithelium remained intact with multi-layers morphology, and maintained the corneal epithelial phenotype with expression of corneal epithelial markers, such as K3, p63, intergrinβ1 and EGFR, as evaluated by HE and immunofluorescent staining (Fig 6). The results suggest that the new corneal constructs may have potential clinical application. Further evaluation is necessary to perform new experiments for transplantation of these corneal construct to rabbit eye.

In conclusion, this study investigated a new substrate, a natural corneal stroma, for bioengineering an artificial corneal construct. Our findings demonstrated that in combination with fresh donor limbal epithelium that contains stem cells, the donor corneal stroma, a great source of natural substrate, is useful to bioengineer a native-like corneal equivalent construct with proliferative potential. This corneal construct provides a new approach to corneal reconstruction.

## Materials and Methods

### Materials and Reagents

Cell culture plates, centrifuge tubes and other plastic ware were purchased from Becton Dickinson (Lincoln Park, NJ). Dulbecco modified Eagle medium (DMEM), Ham F-12, amphotericin B, gentamicin, 0.25% trypsin/0.03% EDTA solution, and antibody against connexin 43 (Cx43) were from Invitrogen-GIBCO (Grand Island, NY). Fetal bovine serum (FBS) was from Hyclone (Logan, UT). Monoclonal antibodies (mAb) against p63, integrin β1 and EGFR came from BioLegend (San Diego, CA). Goat polyclonal antibody against involucrin was from Santa Cruz Biotechnology (Santa Cruz, CA). ABCG2 and K3 mAbs were from Millipore (Billerica, MA). Fluorescein Alexa Fluor 488 conjugated second antibodies (goat anti-mouse or anti-rabbit IgG) were from Molecular Probes (Eugene, OR). Hydrocortisone, human EGF, cholera toxin A subunit, dimethyl sulfoxide (DMSO), Hoechst 33342 and other reagents came from Sigma (St Louis, MO).

### Human Corneal Tissues and Preparation of Stromal Discs

Human corneoscleral tissues that were not suitable for clinical use, from donors aged 19–71 years, were obtained from the Lions Eye Bankof Texas (LEBT, Houston, TX). The details of the donors’ condition, tissue procurement and length of preservation were supplied by the Eye Bank. These tissues were preserved in Optisol™-GS (Bausch and Lomb Inc, Rochester, NY) at 4°C. Donor tissues were handled according to the tenets of the Declaration of Helsinki. Cryosections were prepared for cornel and limbal immunostaining as previously described [19].

A total of 62 corneal stromal lamella discs were prepared from cadaver corneal tissues preserved in Optisol solution for 13–90 days (Fig 1A). After corneal epithelium was gently removed, the anterior corneal stroma was cut to 10–11 mm diameter discs with 200 µm thickness (including basement membrane, Bowman’s layer and anterior part of corneal stroma) by the Horizon Epikeratome system (Fig 1 B–D). One cornea only made one stromal disc. The corneal stroma discs were placed on culture inserts in 12-well plates (Fig 1E). The stromal disc size is designed to be capable of covering the entire surface of recipient cornea when it is used for clinic in the future.

### Preparation of Human Amniotic Membrane and Hydrogel

The protocol for preparation of human amniotic membrane was approved by the Baylor College of Medicine Institutional Review Board. In accordance with the tenets of the Declaration of Helsinki and with the written informed consent from a donor, human amniotic membrane was obtained at the time of elective caesarean sections and processed as previously reported [33]. In brief, the placenta tissue was washed with PBS containing antibiotics, and then stored in DMEM/F-12 containing 50% glycerol at −70°C for up to 3 months, pending testing of donor sera for disease. Just before use, the amniotic membrane was thawed, washed with PBS and cut into pieces of approximately 3 cm in diameter, incubated with 0.25% tripsin-0.03% EDTA at 37°C for 2 hours to loosen cellular adhesion followed by gentle scraping with a cell scraper to remove the epithelium without breaking the basement membrane. The denuded HAM with basement membrane side up was used for human corneal epithelial culture. The hydrogel was prepared using a HyStem-C Hydrogel kit that contains three main components, HyStem, Gelin-S and Extralink, and degassed water to make hydrogel in the culture inserts, according to the manufacturer’s protocol of the kit (Glycosan Biosystems, Salt Lake City, UT).

### Regeneration of Corneal Epithelium on Three Different Substrates from Fresh Human Limbal Explants

Human limbal epithelial cells were cultured using explants from corneal limbal rims by our previous method [26], [34]. In brief, the limbal ring was cut into 12 pieces with similar size of approximately 2 mm×2 mm each. The fresh human limbal explants were cultured onto the basement membrane or stroma side of corneal stromal discs, the denuded amniotic membrane or HyStem-C hydrogel. All cultures were incubated in SHEM medium, consisting of a 1∶1mixture of DMEM and Ham’s F12 medium containing 5 ng/mL EGF, 5 µg/mL insulin, 5 µg/mL transferrin, 5 ng/mL sodium selenite, 0.5 µg/mL hydrocortisone, 30 ng/mL cholera toxin A, 0.5% DMSO, 50 µg/mL gentamicin, 1.25 µg/mL amphotericin B and 5% FBS, at 37°C under 5% CO_2_ and 95% humidity (Fig 1F). The medium was renewed every 2–3 days. The confluent cultures were exposed to air-lift semi-dry conditions by adjusting the medium level for an additional 7 days to form stratified multi-layer corneal epithelial sheets, which was used to prepare cryosections for evaluation.

### Regenerating Capacity of Epithelium in a Wound Healing Model

After a multiple-layer epithelium was generated on human corneal stroma, a 4 mm diameter area was deeply wounded in the center of the epithelium by a 4 mm diameter filter paper soaked with 1 N NaOH for 5–10 seconds. The wound epithelium had been observed for 4–5 days until the wounded area was completely healed by observation under a phase microscope. This wound healing experiment was performed continually once a week for 3 or more times.

### Xeno-transplantation of Bioengineered Corneal Constructs to Nude Mice

The corneal constructs were produced using limbal explants cultivated on the corneal stromal lamella discs. The constructs were transplanted into a small pocket created in skin on the back of NIH bg-nudxidBR nude mice (Charles River Laboratories, Wilmington, MA) after anesthetized with intraperitoneal injection of 0.05 mL/30 g of body weight of “Rodent Combo III” (Ketamine, xylazine and acepromizine) (Fig 1G). These transplants in the nude mice were evaluated in vivo at different timepoints (7 and 14 days). At each timepoint, the mice were euthanized and the transplanted grafts were removed and prepared for cryosections to evaluate their survival, growth and cell phenotype. This animal research protocol was approved by the Center for Comparative Medicine at Baylor College of Medicine. All animals used in this study were treated in accordance with the guidelines provided in the Association for Research in Vision and Ophthalmology statement for the Use of Animals in Ophthalmic and Vision Research.

### Hematoxylin and Eosin (HE) and Immunofluorescent Staining

HE staining and immunofluorescent staining were performed on cryosections from tissues and cultivated constructs using a previously reported method [19], [26]. In brief, after fixed in cold methanol, permeabilized with 0.2% Triton X-100 in PBS for 10 min, and blocked with 5% normal goat serum in PBS for 30 min, the sections were applied with primary antibodies against ABCG2, p63, integrin β1, EGFR, K3, involucrin, connexin 43 or laminin-5, and incubated for 1 hour at room temperature. Secondary antibodies, Alexa Fluor 488 conjugated goat anti-mouse IgG (1∶300), was then applied and incubated in a dark chamber for 1 hour, followed by counterstaining with Hoechst 33342 DNA binding dye (1 µg/mL in PBS) for 5 min. After PBS wash, Antifade Gel/Mount (Fisher, Atlanta, GA) and a cover slip were applied. Sections were examined and photographed with an epifluorescent microscope (Eclipse 400, Nikon, Japan) with a digital camera (model DMX 1200, Nikon).

## References

[1] Dua HS, Saini JS, Azuara-Blanco A, Gupta P (2000) Limbal stem cell deficiency: concept, aetiology, clinical presentation, diagnosis and management. Indian J Ophthalmol 48: 83–92. 11116520

[2] Selvam S, Thomas PB, Yiu SC (2006) Tissue engineering: current and future approaches to ocular surface reconstruction. Ocul Surf 4: 120–136. 16900268 10.1016/s1542-0124(12)70039-3

[3] Germain L, Carrier P, Auger FA, Salesse C, Guerin SL (2000) Can we produce a human corneal equivalent by tissue engineering? Prog Retin Eye Res 19: 497–527. 10925241 10.1016/s1350-9462(00)00005-7

[4] Nishida K (2003) Tissue engineering of the cornea. Cornea 22: S28–S34. 14703705 10.1097/00003226-200310001-00005

[5] Pellegrini G, Traverso CE, Franzi AT, Zingirian M, Cancedda R, et al (1997) Long-term restoration of damaged corneal surfaces with autologous cultivated corneal epithelium. Lancet 349: 990–993. 9100626 10.1016/S0140-6736(96)11188-0

[6] Tsai RJ, Li LM, Chen JK (2000) Reconstruction of damaged corneas by transplantation of autologous limbal epithelial cells. N Engl J Med 343: 86–93. 10891515 10.1056/NEJM200007133430202

[7] Schwab IR, Reyes M, Isseroff RR (2000) Successful transplantation of bioengineered tissue replacements in patients with ocular surface disease. Cornea 19: 421–426. 10928750 10.1097/00003226-200007000-00003

[8] Koizumi N, Inatomi T, Suzuki T, Sotozono C, Kinoshita S (2001) Cultivated corneal epithelial stem cell transplantation in ocular surface disorders. Ophthalmology 108: 1569–1574. 11535452 10.1016/s0161-6420(01)00694-7

[9] Rama P, Bonini S, Lambiase A, Golisano O, Paterna P, et al (2001) Autologous fibrin-cultured limbal stem cells permanently restore the corneal surface of patients with total limbal stem cell deficiency1. Transplantation 72: 1478–1485. 11707733 10.1097/00007890-200111150-00002

[10] Rama P, Matuska S, Paganoni G, Spinelli A, De LM, et al (2010) Limbal stem-cell therapy and long-term corneal regeneration. N Engl J Med 363: 147–155. 20573916 10.1056/NEJMoa0905955

[11] Doillon CJ, Watsky MA, Hakim M, Wang J, Munger R, et al (2003) A collagen-based scaffold for a tissue engineered human cornea: physical and physiological properties. Int J Artif Organs 26: 764–773. 14521175 10.1177/039139880302600810

[12] Gil ES, Mandal BB, Park SH, Marchant JK, Omenetto FG, et al (2010) Helicoidal multi-lamellar features of RGD-functionalized silk biomaterials for corneal tissue engineering. Biomaterials 31: 8953–8963. 20801503 10.1016/j.biomaterials.2010.08.017 PMC2949540

[13] Donohue DJ, Stoyanov BJ, McCally RL, Farrell RA (1995) Numerical modeling of the cornea’s lamellar structure and birefringence properties. J Opt Soc Am A Opt Image Sci Vis 12: 1425–1438. 7608787 10.1364/josaa.12.001425

[14] Jones RR, Hamley IW, Connon CJ (2012) Ex vivo expansion of limbal stem cells is affected by substrate properties. Stem Cell Res 8: 403–409. 22386779 10.1016/j.scr.2012.01.001

[15] Chen B, Jones RR, Mi S, Foster J, Alcock SG, Hamley IW, et al (2012) The mechanical properties of amniotic membrane influence its effect as a biomaterial for ocular surface repair. Soft Matter 8: 8379–8387.

[16] Suzuki K, Saito J, Yanai R, Yamada N, Chikama T, et al (2003) Cell-matrix and cell-cell interactions during corneal epithelial wound healing. Prog Retin Eye Res 22: 113–133. 12604055 10.1016/s1350-9462(02)00042-3

[17] Gipson IK, Spurr-Michaud S, Tisdale A, Keough M (1989) Reassembly of the anchoring structures of the corneal epithelium during wound repair in the rabbit. Invest Ophthalmol Vis Sci 30: 425–434. 2925314

[18] Koizumi N, Fullwood NJ, Bairaktaris G, Inatomi T, Kinoshita S, et al (2000) Cultivation of corneal epithelial cells on intact and denuded human amniotic membrane. Invest Ophthalmol Vis Sci 41: 2506–2513. 10937561

[19] Chen Z, de Paiva CS, Luo L, Kretzer FL, Pflugfelder SC, et al (2004) Characterization of putative stem cell phenotype in human limbal epithelia. Stem Cells 22: 355–366. 15153612 10.1634/stemcells.22-3-355 PMC2906385

[20] Tong L, Corrales RM, Chen Z, Villarreal AL, de Paiva CS, et al (2006) Expression and regulation of cornified envelope proteins in human corneal epithelium. Invest Ophthalmol Vis Sci 47: 1938–1946. 16639001 10.1167/iovs.05-1129 PMC2906387

[21] Chen Z, Evans WH, Pflugfelder SC, Li D-Q (2006) Gap junction protein connexin 43 serves as a negative marker for a stem cell-containing population of human limbal epithelial cells. Stem Cells 24: 1265–1273. 16424398 10.1634/stemcells.2005-0363 PMC2906383

[22] Budak MT, Alpdogan OS, Zhou M, Lavker RM, Akinci MA, et al (2005) Ocular surface epithelia contain ABCG2-dependent side population cells exhibiting features associated with stem cells. J Cell Sci 118: 1715–1724. 15811951 10.1242/jcs.02279 PMC1237017

[23] Schlotzer-Schrehardt U, Kruse FE (2005) Identification and characterization of limbal stem cells. Exp Eye Res 81: 247–264. 16051216 10.1016/j.exer.2005.02.016

[24] Pajoohesh-Ganji A, Stepp MA (2005) In search of markers for the stem cells of the corneal epithelium. Biol Cell 97: 265–276. 15762848 10.1042/BC20040114

[25] Chen B, Mi S, Wright B, Connon CJ (2010) Investigation of K14/K5 as a stem cell marker in the limbal region of the bovine cornea. PLoS One 5: e13192. 10.1371/journal.pone.0013192 [doi]. 10.1371/journal.pone.0013192 PMC2950846 20949137

[26] Kim HS, XJ Song, de Paiva CS, Chen Z, Pflugfelder SC, et al (2004) Phenotypic characterization of human corneal epithelial cells expanded ex vivo from limbal explant and single cell cultures. Exp Eye Res 79: 41–49. 15183099 10.1016/j.exer.2004.02.015 PMC2906376

[27] Li D-Q, Chen Z, Song XJ, de Paiva CS, Kim HS, et al (2005) Partial enrichment of a population of human limbal epithelial cells with putative stem cell properties based on collagen type IV adhesiveness. Exp Eye Res 80: 581–590. 15781286 10.1016/j.exer.2004.11.011 PMC2906384

[28] de Paiva CS, Chen Z, Corrales RM, Pflugfelder SC, Li D-Q (2005) ABCG2 transporter identifies a population of clonogenic human limbal epithelial cells. Stem Cells 23: 63–73. 15625123 10.1634/stemcells.2004-0093 PMC2906389

[29] de Paiva CS, Pflugfelder SC, Li D-Q (2006) Cell size correlates with phenotype and proliferative capacity in human corneal epithelial cells. Stem Cells 24: 368–375. 16123387 10.1634/stemcells.2005-0148 PMC2906390

[30] Qi H, Li DQ, Shine HD, Chen Z, Yoon KC, et al (2008) Nerve growth factor and its receptor TrkA serve as potential markers for human corneal epithelial progenitor cells. Exp Eye Res 86: 34–40. 17980361 10.1016/j.exer.2007.09.003 PMC2198932

[31] Qi H, Shine HD, Li DQ, de Paiva CS, Farley WJ, et al (2008) Glial cell-derived neurotrophic factor gene delivery enhances survival of human corneal epithelium in culture and the overexpression of GDNF in bioengineered constructs. Exp Eye Res 87: 580–586. 18938159 10.1016/j.exer.2008.09.012 PMC4529993

[32] Lu R, Qu Y, Ge J, Zhang L, Su Z, et al (2012) Transcription factor TCF4 maintains the properties of human corneal epithelial stem cells. Stem Cells 30: 753–761. 22232078 10.1002/stem.1032 PMC5610543

[33] Kim JC, Tseng SC (1995) Transplantation of preserved human amniotic membrane for surface reconstruction in severely damaged rabbit corneas. Cornea 14: 473–484. 8536460

[34] Chen Z, Li D-Q, Tong L, Stewart P, Chu C, et al (2006) Targeted inhibition of p57 and p15 blocks transforming growth factor beta-inhibited proliferation of primary cultured human limbal epithelial cells. Mol Vis 12: 983–994. 16943770 PMC2906388

